# 1-[(4-Chloro­phen­yl)(phen­yl)meth­yl]piperazine-1,4-diium bis­(trichloro­acetate)–trichloro­acetic acid (1/1)

**DOI:** 10.1107/S1600536812034794

**Published:** 2012-08-11

**Authors:** Yanxi Song, C. S. Chidan Kumar, Mehmet Akkurt, S. Chandraju, Hongqi Li

**Affiliations:** a School of Environmental Science and Engineering, Donghua University, Shanghai 201620, People’s Repulic of China; b Department of Chemistry, Alva’s Institute of Engineering & Technology, Shobhavana Campus, Mijar, Moodbidri 574 225, South Canara District, Karnataka, India; c Department of Physics, Faculty of Sciences, Erciyes University, 38039 Kayseri, Turkey; d Department of Sugar Technology, University of Mysore, Sir. M.V. PG Center, Tubinakere 571 402, India; e Key Laboratory of Science & Technology of Eco-Textiles, Ministry of Education, College of Chemistry, Chemical Engineering & Biotechnology, Donghua University, Shanghai 201620, People’s Repulic of China

## Abstract

In the title salt adduct, C_17_H_21_ClN_2_^2+^·2C_2_Cl_3_O_2_^−^·C_2_HCl_3_O_2_, the Cl atom of the dication is disordered over two positions in a 0.915 (3):0.085 (3) ratio. The Cl atoms in the trichloroacetate anions and trichloroacetic acid molecule are also disordered, with refined site-occupation factors of 0.59 (3):0.41 (3), 0.503 (12):0.417 (12) and 0.653 (12):0.347 (12). The piperazine ring adopts a chair conformation, with puckering parameters *Q*_T_ = 0.587 (3) Å, θ = 2.6 (2) and Φ 334 (6)°. In the crystal, neighbouring mol­ecules are linked by N—H⋯O, O—H⋯O, N—H⋯Cl, C—H⋯O and C—H⋯Cl hydrogen bonds, forming a three-dimensional network.

## Related literature

For the biological activity of piperazine derivatives, see: Dinsmore *et al.* (2002 ▶); Berkheij *et al.* (2005 ▶); Humle & Cherrier (1999 ▶); Campbell *et al.* (1973 ▶). For related structures, see: Jasinski *et al.* (2011 ▶); Song *et al.* (2012 ▶). For puckering parameters, see: Cremer & Pople (1975 ▶).
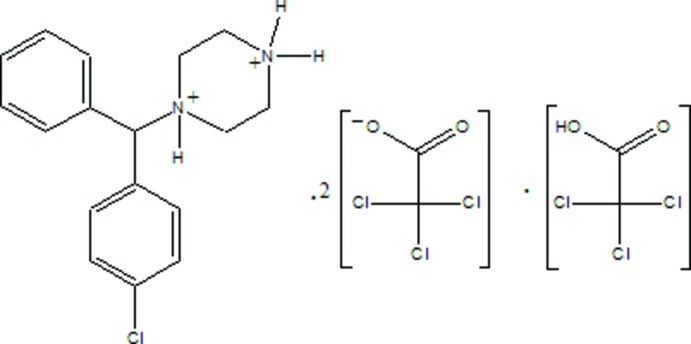


## Experimental

### 

#### Crystal data

C_17_H_21_ClN_2_^2+^·2C_2_Cl_3_O_2_^−^·C_2_HCl_3_O_2_
*M* *_r_* = 776.93
Triclinic, 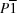

*a* = 9.746 (2) Å
*b* = 13.096 (3) Å
*c* = 13.725 (3) Å
α = 88.317 (3)°
β = 73.127 (3)°
γ = 77.169 (3)°
*V* = 1633.3 (6) Å^3^
*Z* = 2
Mo *K*α radiation
μ = 0.89 mm^−1^
*T* = 293 K
0.27 × 0.22 × 0.15 mm

#### Data collection

Bruker APEXII CCD diffractometer
Absorption correction: multi-scan (*SADABS*; Bruker, 2004) ▶ *T*_min_ = 0.790, *T*_max_ = 0.875
10010 measured reflections
7039 independent reflections
5186 reflections with *I* > 2σ(*I*)
*R*_int_ = 0.012

#### Refinement

*R*[*F* ^2^ > 2σ(*F* ^2^)] = 0.047
*wR*(*F* ^2^) = 0.125
*S* = 1.04
7039 reflections
468 parameters
38 restraints
H atoms treated by a mixture of independent and constrained refinement
Δρ_max_ = 0.40 e Å^−3^
Δρ_min_ = −0.33 e Å^−3^

### 

Data collection: *APEX2* (Bruker, 2004 ▶); cell refinement: *SAINT* (Bruker, 2004 ▶); data reduction: *SAINT*; program(s) used to solve structure: *SIR97* (Altomare *et al.*, 1999 ▶); program(s) used to refine structure: *SHELXL97* (Sheldrick, 2008 ▶); molecular graphics: *ORTEP-3 for Windows* (Farrugia, 2012 ▶); software used to prepare material for publication: *WinGX* (Farrugia, 2012 ▶) and *PLATON* (Spek, 2009 ▶).

## Supplementary Material

Crystal structure: contains datablock(s) I, global. DOI: 10.1107/S1600536812034794/fj2574sup1.cif

Structure factors: contains datablock(s) I. DOI: 10.1107/S1600536812034794/fj2574Isup2.hkl

Supplementary material file. DOI: 10.1107/S1600536812034794/fj2574Isup3.cml

Additional supplementary materials:  crystallographic information; 3D view; checkCIF report

## Figures and Tables

**Table 1:** Hydrogen-bond geometry (Å, °)

*D*—H⋯*A*	*D*—H	H⋯*A*	*D*⋯*A*	*D*—H⋯*A*
N1—H1*A*⋯O3	0.91	1.78	2.685 (3)	172
O1—H1*B*⋯O6^i^	0.82	1.74	2.560 (4)	178
N2—H2*A*⋯Cl6*B* ^ii^	0.90	2.74	3.299 (5)	121
N2—H2*A*⋯O4^ii^	0.90	1.84	2.716 (3)	162
N2—H2*B*⋯O5^iii^	0.90	1.84	2.710 (3)	161
C7—H7⋯O2^iv^	0.98	2.47	3.435 (4)	167
C9—H9⋯O3	0.93	2.39	3.270 (3)	157
C14—H14*B*⋯O6^iii^	0.97	2.42	3.323 (4)	156
C15—H15*B*⋯Cl7*A* ^v^	0.97	2.79	3.526 (5)	133

Symmetry codes: (i) 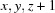; (ii) 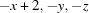; (iii) 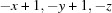; (iv) 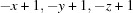; (v) 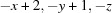.

**Fig. 1.**
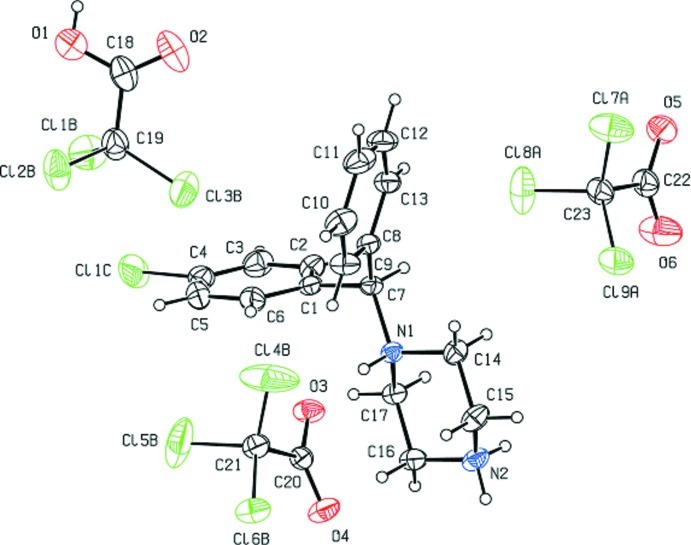
A view of the molecule of the title compound. Displacement ellipsoids are drawn at the 30% probability level and H atoms are omitted for clarity.

**Fig. 2.**
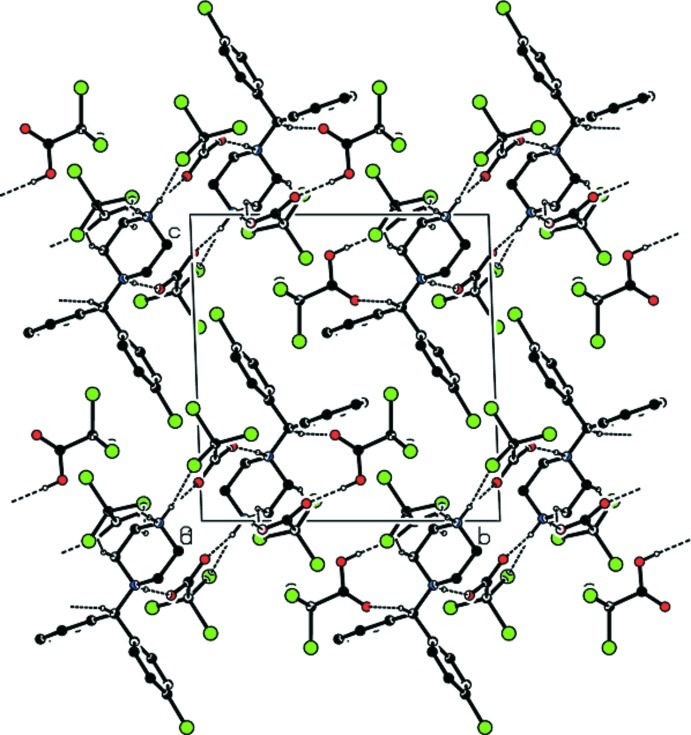
View of the packing and hydroegen bonding diagrams of the title compound along the a axis. H atoms not involved in hydrogen bonding have been omitted for clarity. Only major components of the disorder parts are shown.

## supplementary crystallographic information

### Comment

The piperazine nucleus is capable of binding to multiple receptors with high affinity and therefore piperazine has been classified as a privileged structure (Dinsmore *et al.*, 2002). They are found in biologically active compounds across a number of different therapeutic areas (Berkheij *et al.*, 2005) such as antifungal, antibacterial, antimalarial, antipsychotic, antidepressant and antitumour activity against colon, prostate, breast, lung and leukemia tumors (Humle & Cherrier, 1999). 1-Benzylpiperazine was originally synthesized as a potential antihelminthic (Campbell *et al.*, 1973) and these derivatives were found to possess excellent pharmacological activities such as vasodilator, hypotensive, antiviral activity and cerebral blood flow increasing actions, broad pharmacological action on central nerves system (*CNS*), especially on dopaminergic neurotransmission. In the course of our studies on the salts of piperazines (Jasinski *et al.*, 2011; Song *et al.*, 2012) and in view of the importance of piperazines, the paper reports the crystal and molecular structure of the title salt.

The piperazine ring in the title compound adopts a chair conformation [puckering parameters (Cremer & Pople, 1975) QT = 0.587 (3) Å, θ = 2.6 (2)° and Φ 334 (6)°]. In the crystal, molecules are connected *via* N—H···O, O—H···O, N—H···C, C—H···O and C—H···C hydrogen bonds forming a three dimensional network (Table 1, Fig. 2). Furthermore, C—H···π interactions help to contribute to the stabilization of the structure.

### Experimental

1-((4-Chlorophenyl)(phenyl)methyl)piperazine (2.88 g, 0.01 mol) was dissolved in 10 ml of methanol and trichloroacetic acid (4.89 g, 0.03 mol) was also dissolved in 10 ml of methanol. Both the solutions were mixed and stirred in a beaker over a magnetic plate at 333 K for 30 minutes. The mixture was kept aside for a day at room temperature. The title compound was obtained by the slow evaporation of methanol (m.p: 409 K-411 K).

### Refinement

The hydroxyl H atom appeared in a difference map and was positioned geometrically and refined by using a riding model [O—H = 0.82 Å and *U*_iso_(H) = 1.5Ueq(O)]. The disordered H1C atom attached to C11 was refined with a restrained distance C—H = 0.93 (6) Å and *U*_iso_(H) = 1.2Ueq(C). The rest H atoms bonded to C atoms were located geometrically, with C—H = 0.93–0.98 Å, and refined by using a riding model, with 1.2Ueq(C). The occupancies of the disordered chlorine atoms in three trichloroacetic acid moieties refined to 0.59 (3), 0.41 (3) [for Cl1A,B–Cl3A,B]; 0.503 (12), 0.417 (12) [for Cl4A,B–Cl6A,B] and 0.653 (12), 0.347 (12) [for Cl7A,B–Cl9A,B]. The chlorine atom in the 1-[(4-chlorophenyl)(phenyl)methyl]piperazinediium moiety is disordered on the two-symmetric C atoms of the two benzene rings with refined site-occupation factors of 0.915 (3), 0.085 (3). The disorder was refined using the commands *DFIX* and EADP.

### Crystal data

**Table:**

C_17_H_21_ClN_2_^2+^·2C_2_Cl_3_O_2_^−^·C_2_HCl_3_O_2_	*Z* = 2
*M**_r_* = 776.93	*F*(000) = 784
Triclinic, *P*1	*D*_x_ = 1.580 Mg m^−^^3^
Hall symbol: -P 1	Melting point = 409–411 K
*a* = 9.746 (2) Å	Mo *K*α radiation, λ = 0.71073 Å
*b* = 13.096 (3) Å	Cell parameters from 3555 reflections
*c* = 13.725 (3) Å	θ = 2.2–26.9°
α = 88.317 (3)°	µ = 0.89 mm^−^^1^
β = 73.127 (3)°	*T* = 293 K
γ = 77.169 (3)°	Plate, colourless
*V* = 1633.3 (6) Å^3^	0.27 × 0.22 × 0.15 mm

### Data collection

**Table:**

Bruker APEXII CCD diffractometer	7039 independent reflections
Radiation source: fine-focus sealed tube	5186 reflections with *I* > 2σ(*I*)
Graphite monochromator	*R*_int_ = 0.012
φ and ω scans	θ_max_ = 27.2°, θ_min_ = 2.2°
Absorption correction: multi-scan (*SADABS*; Bruker, 2004)	*h* = −11→12
*T*_min_ = 0.790, *T*_max_ = 0.875	*k* = −16→11
10010 measured reflections	*l* = −16→17

### Refinement

**Table:**

Refinement on *F*^2^	Primary atom site location: structure-invariant direct methods
Least-squares matrix: full	Secondary atom site location: difference Fourier map
*R*[*F*^2^ > 2σ(*F*^2^)] = 0.047	Hydrogen site location: inferred from neighbouring sites
*wR*(*F*^2^) = 0.125	H atoms treated by a mixture of independent and constrained refinement
*S* = 1.04	*w* = 1/[σ^2^(*F*_o_^2^) + (0.0496*P*)^2^ + 0.7913*P*] where *P* = (*F*_o_^2^ + 2*F*_c_^2^)/3
7039 reflections	(Δ/σ)_max_ = 0.001
468 parameters	Δρ_max_ = 0.40 e Å^−^^3^
38 restraints	Δρ_min_ = −0.33 e Å^−^^3^

### Special details

**Table:**

Geometry. Bond distances, angles *etc*. have been calculated using the rounded fractional coordinates. All su's are estimated from the variances of the (full) variance-covariance matrix. The cell e.s.d.'s are taken into account in the estimation of distances, angles and torsion angles
Refinement. Refinement on *F*^2^ for ALL reflections except those flagged by the user for potential systematic errors. Weighted *R*-factors *wR* and all goodnesses of fit *S* are based on *F*^2^, conventional *R*-factors *R* are based on *F*, with *F* set to zero for negative *F*^2^. The observed criterion of *F*^2^ > σ(*F*^2^) is used only for calculating -*R*-factor-obs *etc*. and is not relevant to the choice of reflections for refinement. *R*-factors based on *F*^2^ are statistically about twice as large as those based on *F*, and *R*-factors based on ALL data will be even larger.

### Fractional atomic coordinates and isotropic or equivalent isotropic displacement parameters (Å^2^)

**Table:**

	*x*	*y*	*z*	*U*_iso_*/*U*_eq_	Occ. (<1)
Cl1C	0.31840 (14)	0.07626 (8)	0.67451 (7)	0.1062 (4)	0.915 (3)
N1	0.66237 (18)	0.23191 (14)	0.21015 (13)	0.0366 (5)	
N2	0.7247 (2)	0.14402 (17)	0.00697 (14)	0.0500 (6)	
C1	0.3636 (3)	0.2436 (2)	0.42747 (18)	0.0496 (8)	
C2	0.3041 (3)	0.1922 (2)	0.5133 (2)	0.0623 (10)	
C3	0.3952 (4)	0.1382 (2)	0.5661 (2)	0.0614 (9)	
C4	0.5441 (4)	0.1344 (2)	0.5345 (2)	0.0672 (10)	
C5	0.6024 (3)	0.1857 (2)	0.4488 (2)	0.0545 (8)	
C6	0.5126 (2)	0.24114 (17)	0.39409 (16)	0.0391 (6)	
C7	0.5689 (2)	0.30362 (17)	0.30255 (16)	0.0386 (6)	
C8	0.6491 (3)	0.38233 (17)	0.32521 (16)	0.0417 (7)	
C9	0.7969 (3)	0.3582 (2)	0.3198 (2)	0.0544 (8)	
C10	0.8610 (3)	0.4331 (2)	0.3469 (2)	0.0643 (10)	
C11	0.7786 (4)	0.5318 (3)	0.3801 (2)	0.0690 (11)	
C12	0.6326 (4)	0.5560 (2)	0.3859 (2)	0.0671 (10)	
C13	0.5666 (3)	0.48247 (19)	0.35811 (19)	0.0532 (8)	
C14	0.7043 (3)	0.2947 (2)	0.11745 (17)	0.0472 (8)	
C15	0.8003 (3)	0.2238 (2)	0.02683 (18)	0.0555 (9)	
C16	0.6808 (3)	0.08220 (19)	0.09830 (18)	0.0478 (8)	
C17	0.5831 (2)	0.15348 (18)	0.18780 (17)	0.0416 (7)	
Cl1D	0.8762 (17)	0.5906 (10)	0.4249 (12)	0.123 (6)	0.085 (3)
Cl1B	0.6717 (5)	0.2921 (6)	0.7725 (6)	0.0945 (12)	0.59 (3)
Cl2B	0.9718 (4)	0.2936 (7)	0.7561 (5)	0.0923 (13)	0.59 (3)
Cl3B	0.8561 (7)	0.3498 (10)	0.5860 (4)	0.129 (2)	0.59 (3)
O1	0.7348 (4)	0.46222 (19)	0.8666 (2)	0.0975 (10)	
O2	0.7545 (3)	0.5423 (2)	0.7197 (2)	0.1045 (11)	
C18	0.7641 (3)	0.4663 (3)	0.7690 (3)	0.0725 (11)	
C19	0.8137 (3)	0.3552 (3)	0.7202 (2)	0.0700 (10)	
Cl1A	0.6634 (9)	0.2930 (7)	0.7508 (14)	0.121 (3)	0.41 (3)
Cl2A	0.9600 (10)	0.2728 (12)	0.7519 (6)	0.116 (2)	0.41 (3)
Cl3A	0.8603 (7)	0.3771 (15)	0.5911 (6)	0.104 (3)	0.41 (3)
Cl4B	1.1554 (7)	0.1768 (5)	0.2833 (10)	0.163 (3)	0.503 (12)
Cl5B	1.0936 (7)	−0.0153 (9)	0.3672 (4)	0.146 (3)	0.503 (12)
Cl6B	1.3218 (4)	−0.0180 (4)	0.1815 (4)	0.0598 (10)	0.503 (12)
O3	0.90549 (18)	0.11171 (14)	0.24280 (14)	0.0588 (6)	
O4	1.05566 (19)	−0.00573 (16)	0.12532 (14)	0.0631 (7)	
C20	1.0244 (2)	0.05258 (18)	0.19980 (17)	0.0406 (7)	
C21	1.1464 (3)	0.0551 (2)	0.25207 (19)	0.0537 (8)	
Cl4A	1.1704 (3)	0.1899 (2)	0.2413 (3)	0.0618 (8)	0.497 (12)
Cl5A	1.0917 (8)	0.0294 (5)	0.3806 (3)	0.1017 (17)	0.497 (12)
Cl6A	1.3180 (5)	−0.0238 (5)	0.1946 (6)	0.0861 (16)	0.497 (12)
Cl7A	0.8144 (3)	0.8071 (2)	0.0564 (3)	0.0937 (8)	0.653 (12)
Cl8A	0.7410 (4)	0.6177 (5)	0.1419 (3)	0.1097 (12)	0.653 (12)
Cl9A	0.9218 (3)	0.6188 (3)	−0.0657 (2)	0.0773 (8)	0.653 (12)
O5	0.5411 (2)	0.79751 (16)	0.02795 (15)	0.0696 (7)	
O6	0.6096 (3)	0.6471 (2)	−0.0567 (2)	0.1044 (13)	
C22	0.6253 (3)	0.7137 (2)	−0.0029 (2)	0.0565 (9)	
C23	0.7680 (3)	0.6882 (2)	0.0312 (2)	0.0588 (9)	
Cl7B	0.7846 (14)	0.7855 (7)	0.1020 (12)	0.155 (4)	0.347 (12)
Cl8B	0.7349 (10)	0.5792 (8)	0.1111 (9)	0.134 (3)	0.347 (12)
Cl9B	0.9191 (8)	0.6431 (10)	−0.0692 (6)	0.141 (3)	0.347 (12)
H1	0.30230	0.28060	0.39140	0.0590*	
H1A	0.74610	0.19660	0.22360	0.0440*	
H1C	0.812 (7)	0.587 (4)	0.400 (5)	0.1680*	0.915 (3)
H2	0.20380	0.19420	0.53480	0.0750*	
H2A	0.78510	0.10080	−0.04520	0.0600*	
H2B	0.64430	0.17580	−0.01100	0.0600*	
H4	0.60480	0.09750	0.57100	0.0810*	
H5	0.70280	0.18320	0.42730	0.0650*	
H7	0.48250	0.34370	0.28470	0.0460*	
H9	0.85350	0.29140	0.29790	0.0650*	
H10	0.96050	0.41640	0.34260	0.0770*	
H12	0.57670	0.62280	0.40870	0.0810*	
H13	0.46750	0.50020	0.36150	0.0640*	
H14A	0.75700	0.34490	0.13060	0.0570*	
H14B	0.61620	0.33320	0.10230	0.0570*	
H15A	0.82360	0.26550	−0.03280	0.0670*	
H15B	0.89180	0.18940	0.03990	0.0670*	
H16A	0.76800	0.04370	0.11480	0.0570*	
H16B	0.62870	0.03200	0.08420	0.0570*	
H17A	0.49400	0.18980	0.17250	0.0500*	
H17B	0.55550	0.11210	0.24720	0.0500*	
H1D	0.35640	0.10400	0.62340	0.1680*	0.085 (3)
H1B	0.69470	0.52090	0.89260	0.1460*	

### Atomic displacement parameters (Å^2^)

**Table:**

	*U*^11^	*U*^22^	*U*^33^	*U*^12^	*U*^13^	*U*^23^
Cl1C	0.1343 (10)	0.0734 (6)	0.0679 (6)	−0.0027 (6)	0.0188 (6)	0.0294 (5)
N1	0.0324 (9)	0.0402 (10)	0.0346 (9)	−0.0041 (7)	−0.0085 (7)	−0.0037 (8)
N2	0.0382 (10)	0.0655 (13)	0.0387 (10)	0.0035 (9)	−0.0094 (8)	−0.0153 (10)
C1	0.0481 (13)	0.0562 (15)	0.0428 (13)	−0.0120 (11)	−0.0102 (11)	−0.0012 (11)
C2	0.0619 (17)	0.0661 (18)	0.0532 (16)	−0.0233 (14)	−0.0007 (13)	−0.0032 (14)
C3	0.082 (2)	0.0423 (14)	0.0446 (14)	−0.0083 (13)	0.0010 (13)	0.0017 (11)
C4	0.080 (2)	0.0580 (17)	0.0522 (16)	0.0059 (15)	−0.0186 (15)	0.0086 (13)
C5	0.0497 (14)	0.0539 (15)	0.0510 (14)	0.0012 (11)	−0.0103 (11)	0.0011 (12)
C6	0.0423 (11)	0.0369 (11)	0.0340 (11)	−0.0053 (9)	−0.0068 (9)	−0.0072 (9)
C7	0.0366 (11)	0.0367 (11)	0.0367 (11)	0.0004 (9)	−0.0075 (9)	−0.0056 (9)
C8	0.0485 (13)	0.0367 (12)	0.0354 (11)	−0.0076 (10)	−0.0065 (9)	−0.0036 (9)
C9	0.0510 (14)	0.0475 (14)	0.0624 (16)	−0.0082 (11)	−0.0136 (12)	−0.0141 (12)
C10	0.0616 (17)	0.0729 (19)	0.0616 (17)	−0.0269 (15)	−0.0128 (14)	−0.0095 (15)
C11	0.088 (2)	0.0628 (19)	0.0562 (17)	−0.0337 (17)	−0.0069 (15)	−0.0114 (14)
C12	0.092 (2)	0.0397 (14)	0.0586 (17)	−0.0133 (14)	−0.0047 (15)	−0.0111 (12)
C13	0.0621 (15)	0.0418 (13)	0.0460 (14)	−0.0038 (11)	−0.0060 (12)	−0.0042 (11)
C14	0.0481 (13)	0.0535 (14)	0.0389 (12)	−0.0147 (11)	−0.0083 (10)	0.0018 (11)
C15	0.0471 (14)	0.0763 (18)	0.0388 (13)	−0.0162 (13)	−0.0034 (11)	−0.0066 (12)
C16	0.0431 (12)	0.0468 (13)	0.0516 (14)	−0.0019 (10)	−0.0155 (11)	−0.0127 (11)
C17	0.0404 (11)	0.0413 (12)	0.0419 (12)	−0.0070 (9)	−0.0110 (9)	−0.0054 (10)
Cl1D	0.138 (13)	0.084 (8)	0.122 (11)	−0.057 (8)	0.028 (9)	−0.043 (8)
Cl1B	0.087 (2)	0.092 (2)	0.103 (2)	−0.0406 (17)	−0.0098 (14)	0.0010 (15)
Cl2B	0.0502 (18)	0.125 (3)	0.080 (2)	0.010 (2)	−0.0111 (14)	0.039 (2)
Cl3B	0.166 (5)	0.130 (4)	0.0581 (17)	0.026 (3)	−0.0279 (19)	0.0032 (17)
O1	0.128 (2)	0.0724 (16)	0.0876 (18)	−0.0050 (15)	−0.0374 (16)	0.0014 (13)
O2	0.1013 (19)	0.0807 (17)	0.136 (2)	−0.0134 (14)	−0.0510 (17)	0.0464 (17)
C18	0.0613 (18)	0.073 (2)	0.089 (2)	−0.0147 (15)	−0.0330 (17)	0.0250 (18)
C19	0.0590 (17)	0.078 (2)	0.0604 (17)	0.0002 (15)	−0.0106 (14)	0.0138 (15)
Cl1A	0.121 (4)	0.069 (3)	0.162 (8)	−0.018 (3)	−0.027 (4)	−0.015 (3)
Cl2A	0.114 (5)	0.116 (4)	0.061 (3)	0.062 (4)	−0.003 (3)	0.007 (3)
Cl3A	0.083 (3)	0.146 (7)	0.064 (3)	0.000 (3)	−0.0147 (18)	0.027 (3)
Cl4B	0.124 (3)	0.115 (3)	0.274 (7)	0.028 (2)	−0.125 (4)	−0.117 (3)
Cl5B	0.096 (3)	0.252 (8)	0.0563 (17)	0.034 (4)	−0.0272 (15)	0.028 (3)
Cl6B	0.0251 (13)	0.075 (2)	0.0773 (16)	−0.0025 (13)	−0.0156 (11)	−0.0275 (15)
O3	0.0395 (9)	0.0604 (11)	0.0696 (12)	0.0093 (8)	−0.0186 (8)	−0.0206 (9)
O4	0.0446 (9)	0.0845 (14)	0.0543 (11)	0.0062 (9)	−0.0178 (8)	−0.0291 (10)
C20	0.0333 (11)	0.0433 (12)	0.0435 (12)	−0.0024 (9)	−0.0130 (9)	−0.0005 (10)
C21	0.0441 (13)	0.0605 (16)	0.0550 (15)	0.0020 (11)	−0.0213 (11)	−0.0107 (12)
Cl4A	0.0429 (12)	0.0478 (11)	0.0974 (18)	−0.0109 (7)	−0.0229 (11)	−0.0082 (12)
Cl5A	0.102 (3)	0.165 (4)	0.0475 (15)	−0.038 (3)	−0.0323 (15)	0.0251 (18)
Cl6A	0.060 (2)	0.075 (2)	0.121 (4)	0.0152 (18)	−0.045 (2)	−0.003 (2)
Cl7A	0.0516 (10)	0.0767 (12)	0.146 (2)	−0.0045 (8)	−0.0225 (13)	−0.0295 (13)
Cl8A	0.0871 (13)	0.160 (3)	0.0825 (15)	−0.0239 (17)	−0.0334 (12)	0.0591 (18)
Cl9A	0.0466 (12)	0.0750 (12)	0.0865 (15)	0.0172 (9)	−0.0047 (9)	−0.0106 (9)
O5	0.0487 (10)	0.0748 (13)	0.0735 (13)	0.0149 (10)	−0.0203 (9)	−0.0066 (11)
O6	0.0810 (16)	0.0876 (17)	0.154 (3)	0.0089 (13)	−0.0651 (17)	−0.0407 (17)
C22	0.0419 (13)	0.0611 (17)	0.0591 (16)	0.0008 (12)	−0.0131 (12)	0.0056 (13)
C23	0.0454 (14)	0.0588 (16)	0.0644 (17)	0.0045 (12)	−0.0164 (12)	0.0032 (13)
Cl7B	0.156 (7)	0.118 (5)	0.214 (9)	0.051 (4)	−0.144 (6)	−0.083 (5)
Cl8B	0.139 (4)	0.125 (5)	0.112 (5)	0.020 (3)	−0.039 (3)	0.058 (4)
Cl9B	0.053 (3)	0.189 (7)	0.167 (6)	−0.035 (4)	0.000 (3)	−0.060 (5)

### Geometric parameters (Å, º)

**Table:**

Cl1C—C3	1.728 (3)	C2—C3	1.370 (4)
Cl1D—C11	1.603 (17)	C3—C4	1.379 (6)
Cl1A—C19	1.772 (11)	C4—C5	1.376 (4)
Cl1B—C19	1.735 (7)	C5—C6	1.387 (4)
Cl2A—C19	1.742 (13)	C6—C7	1.513 (3)
Cl2B—C19	1.770 (7)	C7—C8	1.513 (3)
Cl3A—C19	1.729 (9)	C8—C9	1.385 (4)
Cl3B—C19	1.767 (6)	C8—C13	1.388 (3)
Cl4A—C21	1.828 (4)	C9—C10	1.385 (4)
Cl4B—C21	1.690 (8)	C10—C11	1.373 (5)
Cl5A—C21	1.735 (5)	C11—C12	1.366 (6)
Cl5B—C21	1.807 (8)	C12—C13	1.388 (4)
Cl6A—C21	1.732 (7)	C14—C15	1.514 (3)
Cl6B—C21	1.773 (6)	C16—C17	1.510 (3)
Cl7A—C23	1.784 (4)	C1—H1	0.9300
Cl7B—C23	1.688 (13)	C2—H2	0.9300
Cl8A—C23	1.739 (5)	C3—H1D	0.9200
Cl8B—C23	1.803 (11)	C4—H4	0.9300
Cl9A—C23	1.775 (4)	C5—H5	0.9300
Cl9B—C23	1.703 (9)	C7—H7	0.9800
O1—C18	1.288 (5)	C9—H9	0.9300
O2—C18	1.189 (5)	C10—H10	0.9300
O1—H1B	0.8200	C11—H1C	0.93 (6)
O3—C20	1.234 (3)	C12—H12	0.9300
O4—C20	1.218 (3)	C13—H13	0.9300
O5—C22	1.218 (3)	C14—H14B	0.9700
O6—C22	1.222 (4)	C14—H14A	0.9700
N1—C17	1.502 (3)	C15—H15B	0.9700
N1—C14	1.499 (3)	C15—H15A	0.9700
N1—C7	1.530 (3)	C16—H16A	0.9700
N2—C16	1.481 (3)	C16—H16B	0.9700
N2—C15	1.478 (4)	C17—H17B	0.9700
N1—H1A	0.9100	C17—H17A	0.9700
N2—H2B	0.9000	C18—C19	1.535 (5)
N2—H2A	0.9000	C20—C21	1.563 (4)
C1—C2	1.381 (4)	C22—C23	1.558 (4)
C1—C6	1.383 (4)		
			
C18—O1—H1B	110.00	C15—C14—H14A	109.00
C7—N1—C17	111.81 (16)	C15—C14—H14B	110.00
C14—N1—C17	108.77 (17)	N1—C14—H14B	110.00
C7—N1—C14	110.75 (17)	H14A—C14—H14B	108.00
C15—N2—C16	111.02 (19)	N2—C15—H15B	110.00
C17—N1—H1A	109.00	C14—C15—H15A	109.00
C7—N1—H1A	108.00	C14—C15—H15B	110.00
C14—N1—H1A	108.00	H15A—C15—H15B	108.00
C16—N2—H2A	109.00	N2—C15—H15A	110.00
C16—N2—H2B	109.00	N2—C16—H16B	110.00
H2A—N2—H2B	108.00	C17—C16—H16A	110.00
C15—N2—H2B	109.00	N2—C16—H16A	110.00
C15—N2—H2A	109.00	H16A—C16—H16B	108.00
C2—C1—C6	121.3 (3)	C17—C16—H16B	110.00
C1—C2—C3	119.0 (3)	N1—C17—H17A	110.00
C2—C3—C4	121.0 (3)	N1—C17—H17B	110.00
Cl1C—C3—C4	120.7 (2)	C16—C17—H17B	110.00
Cl1C—C3—C2	118.3 (3)	H17A—C17—H17B	108.00
C3—C4—C5	119.6 (3)	C16—C17—H17A	110.00
C4—C5—C6	120.5 (3)	O1—C18—C19	110.2 (3)
C1—C6—C5	118.6 (2)	O2—C18—C19	122.1 (3)
C1—C6—C7	118.4 (2)	O1—C18—O2	127.6 (4)
C5—C6—C7	123.0 (2)	Cl1B—C19—Cl2B	110.1 (4)
N1—C7—C6	111.45 (17)	Cl1B—C19—Cl3B	109.5 (4)
C6—C7—C8	112.71 (17)	Cl2B—C19—Cl3B	109.3 (4)
N1—C7—C8	111.75 (17)	Cl2B—C19—C18	106.4 (3)
C7—C8—C9	123.6 (2)	Cl1B—C19—C18	106.7 (3)
C9—C8—C13	119.0 (2)	Cl1A—C19—C18	109.9 (5)
C7—C8—C13	117.3 (3)	Cl2A—C19—C18	116.1 (5)
C8—C9—C10	120.3 (2)	Cl3A—C19—C18	103.2 (7)
C9—C10—C11	120.4 (3)	Cl1A—C19—Cl2A	108.3 (6)
Cl1D—C11—C12	131.9 (6)	Cl1A—C19—Cl3A	108.3 (7)
Cl1D—C11—C10	107.6 (6)	Cl2A—C19—Cl3A	110.7 (5)
C10—C11—C12	119.5 (3)	Cl3B—C19—C18	114.8 (5)
C11—C12—C13	121.0 (3)	O3—C20—O4	128.7 (2)
C8—C13—C12	119.8 (3)	O3—C20—C21	113.0 (2)
N1—C14—C15	110.6 (2)	O4—C20—C21	118.3 (2)
N2—C15—C14	110.7 (2)	Cl4B—C21—Cl5B	109.1 (5)
N2—C16—C17	110.42 (19)	Cl4B—C21—Cl6B	111.0 (4)
N1—C17—C16	110.10 (19)	Cl4B—C21—C20	114.3 (3)
C2—C1—H1	119.00	Cl5B—C21—Cl6B	105.5 (4)
C6—C1—H1	119.00	Cl5B—C21—C20	103.6 (3)
C3—C2—H2	121.00	Cl6B—C21—C20	112.6 (2)
C1—C2—H2	120.00	Cl4A—C21—C20	104.9 (2)
C2—C3—H1D	119.00	Cl5A—C21—C20	112.1 (3)
C4—C3—H1D	120.00	Cl6A—C21—C20	115.9 (3)
C5—C4—H4	120.00	Cl4A—C21—Cl5A	107.8 (3)
C3—C4—H4	120.00	Cl4A—C21—Cl6A	106.1 (3)
C4—C5—H5	120.00	Cl5A—C21—Cl6A	109.6 (4)
C6—C5—H5	120.00	O5—C22—O6	127.3 (3)
N1—C7—H7	107.00	O5—C22—C23	116.3 (2)
C6—C7—H7	107.00	O6—C22—C23	116.5 (3)
C8—C7—H7	107.00	Cl7A—C23—Cl8A	108.5 (3)
C10—C9—H9	120.00	Cl7A—C23—Cl9A	105.8 (2)
C8—C9—H9	120.00	Cl7A—C23—C22	109.7 (2)
C9—C10—H10	120.00	Cl8A—C23—Cl9A	110.3 (3)
C11—C10—H10	120.00	Cl8A—C23—C22	110.3 (2)
C12—C11—H1C	114 (4)	Cl9A—C23—C22	112.1 (2)
C10—C11—H1C	127 (4)	Cl7B—C23—C22	113.7 (5)
C11—C12—H12	119.00	Cl8B—C23—C22	100.1 (4)
C13—C12—H12	120.00	Cl9B—C23—C22	111.2 (3)
C12—C13—H13	120.00	Cl7B—C23—Cl8B	108.9 (6)
C8—C13—H13	120.00	Cl7B—C23—Cl9B	114.1 (6)
N1—C14—H14A	110.00	Cl8B—C23—Cl9B	107.7 (6)
			
C17—N1—C14—C15	−58.3 (3)	C9—C8—C13—C12	−0.7 (4)
C14—N1—C7—C8	−58.7 (2)	C7—C8—C13—C12	176.1 (2)
C17—N1—C7—C8	179.79 (17)	C13—C8—C9—C10	0.0 (4)
C14—N1—C17—C16	59.2 (2)	C7—C8—C9—C10	−176.6 (2)
C14—N1—C7—C6	174.17 (19)	C8—C9—C10—C11	0.5 (4)
C7—N1—C17—C16	−178.22 (18)	C9—C10—C11—C12	−0.3 (4)
C7—N1—C14—C15	178.4 (2)	C10—C11—C12—C13	−0.4 (4)
C17—N1—C7—C6	52.7 (2)	C11—C12—C13—C8	0.9 (4)
C16—N2—C15—C14	−56.4 (3)	N1—C14—C15—N2	57.3 (3)
C15—N2—C16—C17	57.4 (3)	N2—C16—C17—N1	−59.0 (3)
C2—C1—C6—C7	−177.0 (2)	O1—C18—C19—Cl1B	58.1 (4)
C6—C1—C2—C3	0.2 (4)	O1—C18—C19—Cl2B	−59.4 (4)
C2—C1—C6—C5	−0.1 (4)	O1—C18—C19—Cl3B	179.6 (4)
C1—C2—C3—Cl1C	178.9 (2)	O2—C18—C19—Cl1B	−121.0 (4)
C1—C2—C3—C4	−0.2 (4)	O2—C18—C19—Cl2B	121.5 (4)
Cl1C—C3—C4—C5	−179.0 (2)	O2—C18—C19—Cl3B	0.4 (5)
C2—C3—C4—C5	0.1 (4)	O3—C20—C21—Cl4B	−45.2 (5)
C3—C4—C5—C6	0.0 (4)	O3—C20—C21—Cl5B	73.4 (4)
C4—C5—C6—C1	−0.1 (4)	O3—C20—C21—Cl6B	−173.0 (2)
C4—C5—C6—C7	176.8 (2)	O4—C20—C21—Cl4B	135.6 (5)
C5—C6—C7—C8	−54.2 (3)	O4—C20—C21—Cl5B	−105.8 (4)
C1—C6—C7—C8	122.6 (2)	O4—C20—C21—Cl6B	7.7 (3)
C5—C6—C7—N1	72.4 (3)	O5—C22—C23—Cl7A	−26.7 (3)
C1—C6—C7—N1	−110.8 (2)	O5—C22—C23—Cl8A	92.7 (3)
C6—C7—C8—C9	85.5 (3)	O5—C22—C23—Cl9A	−144.0 (2)
N1—C7—C8—C9	−40.9 (3)	O6—C22—C23—Cl7A	154.0 (3)
N1—C7—C8—C13	142.5 (2)	O6—C22—C23—Cl8A	−86.7 (3)
C6—C7—C8—C13	−91.1 (2)	O6—C22—C23—Cl9A	36.7 (3)

### Hydrogen-bond geometry (Å, º)

**Table:**

*D*—H···*A*	*D*—H	H···*A*	*D*···*A*	*D*—H···*A*
N1—H1*A*···O3	0.91	1.78	2.685 (3)	172
O1—H1*B*···O6^i^	0.82	1.74	2.560 (4)	178
N2—H2*A*···Cl6*B*^ii^	0.90	2.74	3.299 (5)	121
N2—H2*A*···O4^ii^	0.90	1.84	2.716 (3)	162
N2—H2*B*···O5^iii^	0.90	1.84	2.710 (3)	161
C7—H7···O2^iv^	0.98	2.47	3.435 (4)	167
C9—H9···O3	0.93	2.39	3.270 (3)	157
C14—H14*B*···O6^iii^	0.97	2.42	3.323 (4)	156
C15—H15*B*···Cl7*A*^v^	0.97	2.79	3.526 (5)	133

Symmetry codes: (i) *x*, *y*, *z*+1; (ii) −*x*+2, −*y*, −*z*; (iii) −*x*+1, −*y*+1, −*z*; (iv) −*x*+1, −*y*+1, −*z*+1; (v) −*x*+2, −*y*+1, −*z*.

## References

[bb1] Altomare, A., Burla, M. C., Camalli, M., Cascarano, G. L., Giacovazzo, C., Guagliardi, A., Moliterni, A. G. G., Polidori, G. & Spagna, R. (1999). *J. Appl. Cryst.* **32**, 115–119.

[bb2] Berkheij, M., van der Sluis, L., Sewing, C., den Boer, D. J., Terpstra, J. W., Heimstra, H., Bakker, W. I. I., van den Hoogen Band, A. & van Maarseveen, J. H. (2005). *Tetrahedron*, **46**, 2369–2371.

[bb3] Bruker (2004). *APEX2*, *SAINT* and *SADABS* Bruker AXS Inc., Madison, Wisconsin, USA.

[bb4] Campbell, H., Cline, W., Evans, M., Lloyd, J. & Peck, A. W. (1973). *Eur. J. Clin. Pharmacol.* **6**, 170–176. 10.1007/BF00558281 4586849

[bb5] Cremer, D. & Pople, J. A. (1975). *J. Am. Chem. Soc.* **97**, 1354–1358.

[bb6] Dinsmore, C. J. & Beshore, D. C. (2002). *Tetrahedron*, **58**, 3297–3312.

[bb7] Farrugia, L. J. (2012). *J. Appl. Cryst.* **45**, 849–854.

[bb8] Humle, C. & Cherrier, M. P. (1999). *Tetrahedron Lett.* **40**, 5295–5299.

[bb9] Jasinski, J. P., Butcher, R. J., Siddegowda, M. S., Yathirajan, H. S. & Chidan Kumar, C. S. (2011). *Acta Cryst.* E**67**, o500–o501. 10.1107/S1600536811002674 PMC3051536 21523154

[bb10] Sheldrick, G. M. (2008). *Acta Cryst.* A**64**, 112–122. 10.1107/S0108767307043930 18156677

[bb11] Song, Y., Chidan Kumar, C. S., Nethravathi, G. B., Naveen, S. & Li, H. (2012). *Acta Cryst.* E**68**, o1747. 10.1107/S1600536812020764 PMC3379334 22719532

[bb12] Spek, A. L. (2009). *Acta Cryst.* D**65**, 148–155. 10.1107/S090744490804362X PMC2631630 19171970

